# Gamma-Aminobutyric Acid (GABA) Inhibits α-Melanocyte-Stimulating Hormone-Induced Melanogenesis through GABA_A_ and GABA_B_ Receptors

**DOI:** 10.3390/ijms22158257

**Published:** 2021-07-31

**Authors:** Ilandarage Menu Neelaka Molagoda, Mirissa Hewage Dumindu Kavinda, Hyung Won Ryu, Yung Hyun Choi, Jin-Woo Jeong, Sanghyuck Kang, Gi-Young Kim

**Affiliations:** 1 Department of Marine Life Science, Jeju National University, Jeju 63243, Korea; neelakagm2012@gmail.com (I.M.N.M.); dumindu.kaviya@gmail.com (M.H.D.K.); 2 Research Institute for Basic Sciences, Jeju National University, Jeju 63243, Korea; 3 Natural Medicine Research Center, Korea Research Institute of Bioscience and Biotechnology, Cheongju 28116, Korea; ryuhw@kribb.re.kr; 4 Department of Biochemistry, College of Oriental Medicine, Dong-Eui University, Busan 47227, Korea; choiyh@deu.ac.kr; 5 Nakdonggang National Institute of Biological Resources, Sangju 37242, Korea; jwjeong@nnibr.re.kr; 6 Korea Beauty Industry Development Institute, Jeju 63243, Korea; ksangh@kbidi.or.kr

**Keywords:** GABA, GABA receptor, melanogenesis, Ca^2+^

## Abstract

Gamma-aminobutyric acid (GABA) is considered the primary inhibitory neurotransmitter in the human cortex. However, whether GABA regulates melanogenesis has not been comprehensively elucidated. In this study, we reveal that GABA (20 mM) significantly inhibited α-melanocyte-stimulating hormone (α-MSH)-induced extracellular (from 354.9% ± 28.4% to 126.5% ± 16.0%) and intracellular melanin contents (from 236.7% ± 11.1% to 102.7% ± 23.1%) in B16F10 melanoma cells, without inducing cytotoxicity. In addition, α-MSH-induced hyperpigmentation in zebrafish larvae was inhibited from 246.3% ± 5.4% to 116.3% ± 3.1% at 40 mM GABA, displaying no apparent cardiotoxicity. We also clarify that the GABA-mediated antimelanogenic properties were related to the direct inhibition of microphthalmia-associated transcription factor (*MITF*) and *tyrosinase* expression by inhibiting cyclic adenosine monophosphate (cAMP) and cAMP response element-binding protein (CREB). Furthermore, under α-MSH stimulation, GABA-related antimelanogenic effects were mediated through the GABA_A_ and GABA_B_ receptors, with subsequent inhibition of Ca^2+^ accumulation. In B16F10 melanoma cells and zebrafish larvae, pretreatment with bicuculline, a GABA_A_ receptor antagonist, and CGP 46381, a GABA_B_ receptor antagonist, reversed the antimelanogenic effect of GABA following α-MSH treatment by upregulating Ca^2+^ accumulation. In conclusion, our results indicate that GABA inhibits α-MSH-induced melanogenesis. Hence, in addition to the health benefits of GABA in the central nervous system, it could ameliorate hyperpigmentation disorders.

## 1. Introduction

Melanin is a primary determinant of skin color that protects the skin from adverse external stimuli such as ultraviolet (UV) radiation [1]. In response to UV radiation, α-melanocyte-stimulating hormone (α-MSH) is produced from the skin and pituitary gland, which mediates the onset of melanin production in melanocytes [2]. Subsequently, α-MSH binds to the melanocortin 1 receptor (MC1R), stimulating the downstream signaling pathways through adenylyl cyclase (AC). In particular, AC catalyzes the conversion of adenosine triphosphate (ATP) to cyclic adenosine monophosphate (cAMP), and, subsequently, the protein kinase A (PKA)–cAMP response element-binding protein (CREB) axis, which is mainly considered to promote melanogenesis through the transactivation of microphthalmia-associated transcription factor (*MITF*) [3]. In addition, the binding of α-MSH to MC1R is responsible for initiating store-operated Ca^2+^ entry pathways via Ca^2+^ release from the endoplasmic reticulum (ER) lumen into the cytosol [4]. Changes in cytosolic Ca^2+^ levels are associated with melanin biosynthesis through modulation of AC [5]. Finally, *MITF* translocates to the nucleus and promotes the transcriptional upregulation of melanogenic enzymes such as *tyrosinase* and *tyrosinase*-related protein-1/2 [6]. Nevertheless, excessive melanin production can induce several diseases, including melasma, post-inflammatory hyperpigmentation, and cutaneous amyloidosis [7].

Gamma-aminobutyric acid (GABA) is widely distributed in the cortical neurons and reduces neuronal excitability in the cortex through a decrease in the neuronal action potential [8]. Hence, it is well known that reduced GABA levels are associated with various neuronal disorders, such as anxiety, depression, insomnia, and epilepsy, thus indicating that GABA regulates neuronal disorders [9]. In addition, GABA supplementation has been markedly effective against cardiovascular diseases [10,11], type 1 diabetes [12], and alcoholism [13] through specific GABA receptors. Several different GABA receptors are available for the orthosteric binding of GABA, namely, the GABA_A,_ GABA_B_, and GABA_C_ (a subtype of GABA_A_) receptors [14,15]. The GABA_A_ and GABA_C_ receptors are heteropentameric ligand-gated ion channels with three different subunits [16] and are known to facilitate the allosteric binding of benzodiazepines [17]. There are 16 genes responsible for the synthesis of GABA_A_ receptors, including several subunits, such as α1-6, β1-3, γ1-3, δ, ε, θ, and π, while three genes are responsible for the synthesis of rho (ρ) subunits of GABA_C_ (a subclass of the GABA_A_ receptor) [18]. Allosteric and orthosteric activation of GABA_A_ and GABA_c_ stimulates chloride channels associated with the modulation of anxiety, vigilance, memory, and learning [19,20]. Unlike GABA_A_ and GABA_C_, GABA_B_ is a metabotropic guanine nucleotide-binding protein couple receptor (GPCR) comprising two subunits, GABA_B1_ and GABA_B2_. Activation of GABA_B_ is considered to demonstrate an inhibitory action due to the coupling with inhibitory G_α_ (G_αi/o_), inwardly rectifying the potassium channels and inactivating Ca^2+^ channels, which, in turn, inhibits AC activation [21]. Although the pharmacological benefits of GABA have been extensively identified, it remains unclear which GABA receptor is related to melanin biosynthesis.

In the present study, we evaluated the antimelanogenic properties of GABA in α-MSH-stimulated conditions. Herein, we observed that GABA effectively inhibited α-MSH-stimulated hyperpigmentation in B16F10 melanoma cells and zebrafish larvae by inhibiting intracellular Ca^2+^, which, in turn, inhibited the cAMP–CREB–*MITF*–*tyrosinase* axis. Furthermore, the GABA_A_ and GABA_B_ receptors were found to be responsible for the antimelanogenic properties of GABA.

## 2. Results

### 2.1. Low Concentrations of GABA Present No Cytotoxicity in B16F10 Melanoma Cells

**Figure 1:**
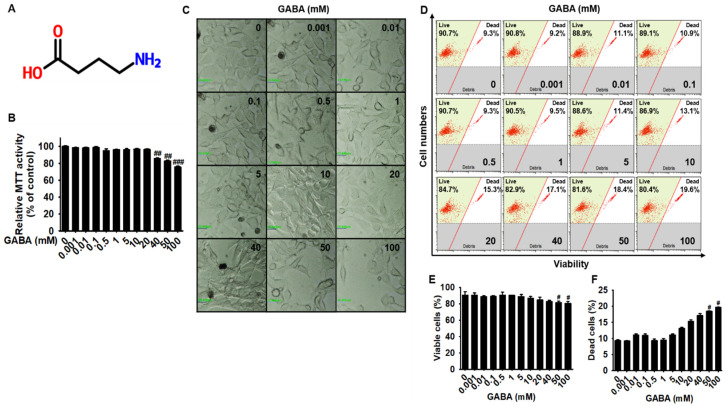
Low concentrations of GABA show no cytotoxicity in B16F10 melanoma cells. B16F10 melanoma cells (5 × 10^4^ cells/mL) were treated with the indicated concentrations of GABA (0–100 mM) for 96 h. (**A**) Chemical structure of GABA. (**B**) Cell viability was measured using an MTT assay. (**C**) Cellular morphology was observed using phase-contrast microscopy (×20). Scale bars = 20 µm. (**D**) Cell viability and dead cell populations were measured using Muse Cell Viability kit. Viable and (**E**) dead cell (**F**) populations are shown. Significant differences among the groups were determined using one-way ANOVA followed by Bonferroni correction. All data are presented as the standard error of the median from three independent experiments (^###^ *p* < 0.001, ^##^ *p* < 0.01, and ^#^ *p* < 0.05 vs. the untreated cells). GABA, gamma-aminobutyric acid.

To evaluate the cytotoxic effect of GABA (Figure 1A) in B16F10 melanoma cells, the cells were treated with the indicated concentrations of GABA (0–100 mM) for 96 h, and cell viability was examined. As shown in Figure 1B, at concentrations over 40 mM, GABA significantly decreased cell viability (85.6% ± 0.8%, 82.7% ± 0.9%, and 75.5% ± 1.1% at 40, 50, and 100 mM GABA, respectively). Nevertheless, the concentrations below 20 mM appeared to be slightly toxic to cells, and no significant cytotoxicity was observed. Based on microscopy, significant cell debris or floating cells were not observed at concentrations below 40 mM GABA; however, 50 mM GABA considerably decreased cell growth, and 100 mM GABA induced a few shrunken cells (Figure 1C). Then, to further evaluate the cytotoxicity of GABA, the population of viable and dead cells was measured using flow cytometry (Figure 1D). GABA induced a significant decrease in the viable cell population to 81.6% ± 1.8% and 80.4% ± 2.7% at 50 and 100 mM, respectively, and increased the population of dead cells to 18.4% ± 0.14% and 19.6% ± 0.1% at 50 and 100 mM, respectively (Figure 1D). Furthermore, slight changes in the viable and dead cell populations were observed at concentrations of 40 mM GABA; however, the effect was not significant when compared with that in untreated cells. Collectively, these results suggest that low concentrations of GABA (below 20 mM) possess neither cytotoxicity nor anti-proliferative effects in B16F10 melanoma cells.

### 2.2. GABA Inhibits α-MSH-Induced Melanogenesis in B16F10 Melanoma Cells

**Figure 2:**
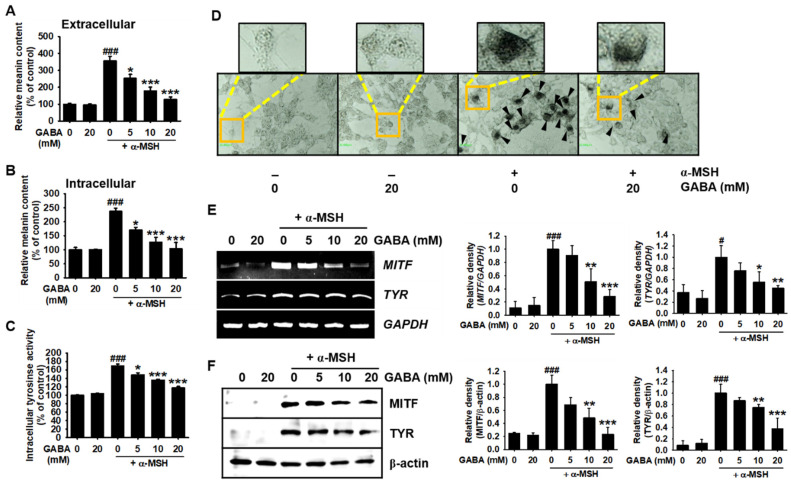
GABA inhibits α-MSH-induced melanogenesis in B16F10 melanoma cells. B16F10 melanoma cells (5 × 10^4^ cells/mL) were treated with the indicated concentrations of GABA (0–20 mM) in the presence and absence of 500 ng/mL α-MSH. After treatment for 96 h, cell-free culture media were collected to measure the extracellular melanin content, and cell pellets were dissolved in 400 μL of 1 M NaOH containing 10% dimethyl sulfoxide (DMSO) at 90°C for 60 min. Absorbance was measured at 405 nm to quantify both (**A**) extracellular and (**B**) intracellular melanin contents. (**C**) The cells were stained for measuring the intracellular *tyrosinase* activity, and (**D**) the images of the cells were captured using phase-contrast microscopy (10×). Scale bars = 20 μm. In a parallel experiment, *MITF* and *tyrosinase* expression were evaluated by (**E**) RT-PCR at 48 h and (**F**) Western blotting at 72 h after chemical treatment. *GAPDH* and β-actin were used as internal controls for RT-PCR and Western blotting, respectively, and the relative expression values were determined (right). The results represent the average of data obtained from three independent experiments and are expressed as the mean ± standard error of the mean (SEM) (*^###^ p* < 0.001 and ^#^ *p* < 0.05 vs. the untreated group; *** *p* < 0.001, ** *p* < 0.01, and * *p* < 0.05 vs. α-MSH-treated cells). GABA, gamma-aminobutyric acid; α-MSH, α-melanocyte-stimulating hormone; RT-PCR, reverse transcription-polymerase chain reaction; *MITF*, microphthalmia-associated transcription factor.

Next, we attempted to evaluate the antimelanogenic effects of GABA. Accordingly, B16F10 melanoma cells were treated with the indicated concentrations of GABA (0–20 mM) in the presence or absence of α-MSH for 96 h, and intracellular and extracellular melanin contents were quantified. α-MSH treatment significantly upregulated the extracellular (Figure 2A) and intracellular melanin (Figure 2B) contents to 354.9% ± 28.4% and 236.7% ± 11.1%, respectively, when compared with those observed in untreated cells. Treatment with GABA reduced the α-MSH-induced melanin contents in a concentration-dependent manner (252.2% ± 23.6%, 178.0% ± 22.5%, and 126.5% ± 16.0% for extracellular melanin contents, and 170.1 ± 19.0%, 127.0 ± 17.2%, and 102.7 ± 23.1% for intracellular melanin contents, at 5, 10, and 20 mM, respectively). Furthermore, α-MSH increased intracellular *tyrosinase* enzyme activity to 169.4 ± 4.2% when compared with that in the untreated cells, which was significantly inhibited following treatment with GABA in a concentration-dependent manner (148.1 ± 4.4%, 135.5 ± 2.1%, and 117.4 ± 3.4% at 5, 10, and 20 mM, respectively, Figure 2C). Consistent with the findings on *tyrosinase* activity, intracellular melanin staining revealed that α-MSH-treated cells contained higher melanin intensities than untreated cells, whereas GABA (20 mM) potently inhibited the high melanin intensity (Figure 2D). We also confirmed that GABA inhibited α-MSH-induced *MITF* and *tyrosinase* expression in a concentration-dependent manner, at both the transcriptional (Figure 2E) and translational (Figure 2F) levels. These results indicate that GABA inhibits melanogenesis in B16F10 melanoma cells.

### 2.3. GABA Inhibits Melanogenesis in Zebrafish Larvae

**Figure 3:**
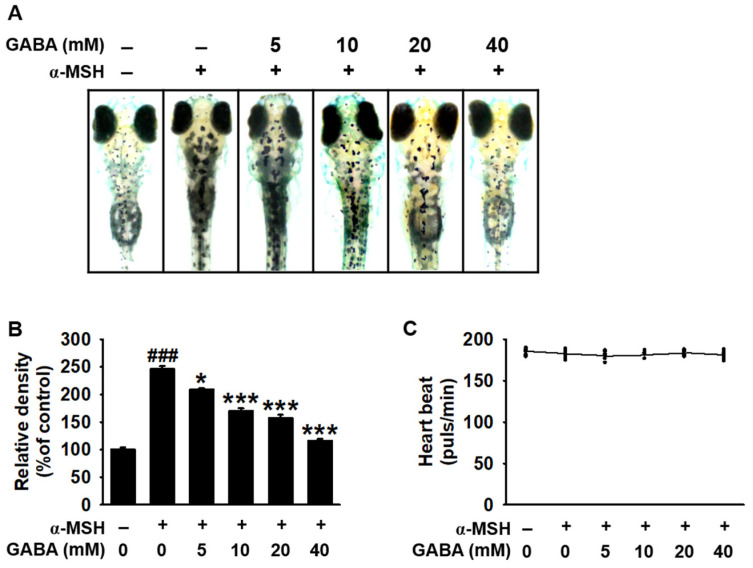
GABA inhibits melanogenesis in zebrafish larvae. Two days post-fertilization (dpf), zebrafish larvae were pretreated with 0.003% PTU for 24 h and then treated at 3 dpf with the indicated concentrations of GABA (0–50 mM) in the presence and absence of 1 µg/mL α-MSH for an additional 72 h. (**A**) Images of zebrafish larvae were captured using a stereomicroscope (4×). (**B**) Melanin densities were quantified using ImageJ software and are expressed as a percentage compared with the untreated larvae. (**C**) The heart rate was assessed to evaluate the cardiotoxicity and is represented as heartbeats/min. The results represent the average of data obtained from three independent experiments and are expressed as the mean ± standard error of the mean (SEM) (^###^ *p* < 0.001 vs. untreated larvae; *** *p* < 0.001 and * *p* < 0.05 vs. α-MSH-treated larvae). GABA, gamma-aminobutyric acid; α-MSH, α-melanocyte-stimulating hormone.

We evaluated the antimelanogenic effect of GABA in vivo. Accordingly, zebrafish larvae (after 3 days post-fertilization (dpf)) were treated with the indicated concentrations of GABA (0–50 mM) in the presence or absence of α-MSH. We observed that α-MSH treatment significantly increased pigmentation in zebrafish larvae to 246.3% ± 5.4% when compared with untreated larvae (Figure 3A,B). However, treatment with GABA decreased the α-MSH-induced melanin content in a concentration-dependent manner (208.9% ± 2.7%, 170.6% ± 4.4%, 157.4% ± 5.8%, and 116.3% ± 3.1% at 5, 10, 20, and 40 mM, respectively; Figure 3B). On evaluating GABA-induced cardiotoxicity by employing the heart rate, we observed that GABA sustained heart rates similar to those observed in untreated larvae (182.3 ± 4.2 beats/min; Figure 3C), indicating that GABA concentrations below 40 mM did not induce any significant cardiotoxicity in zebrafish larvae. Our results suggest that GABA inhibits melanogenesis in zebrafish larvae without any cardiotoxicity.

### 2.4. GABA Inhibits α-MSH-Induced Melanogenesis by Downregulating the cAMP–CREB Axis and Ca^2+^ Levels

**Figure 4:**
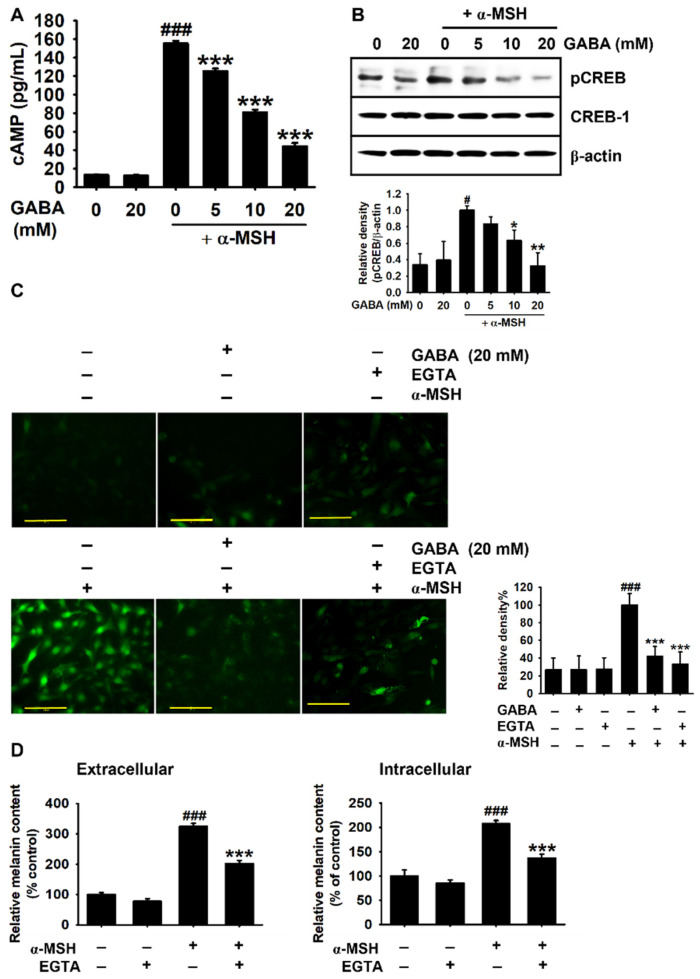
GABA inhibits melanogenesis by downregulating cAMP–CREB-dependent intracellular Ca^2+^. B16F10 melanoma cells (5 × 10^4^ cells/mL) were treated with the indicated concentrations of GABA (0–20 mM) in the presence and absence of 500 ng/mL α-MSH. (**A**) cAMP levels were measured using an ELISA kit 15 min after treatment with GABA (0–20 mM) in the presence and absence of 500 ng/mL α-MSH. (**B**) Total CREB and phosphorylated CREB (pCREB) were evaluated by Western blotting 30 min after GABA treatment. (**C**) The cells were grown in 8-well chamber slides and treated with GABA or 2 mM EGTA in the presence and absence of 500 ng/mL α-MSH for 1 h. The cells were probed with 1 μM Fluo-4 AM for 10 min. Cell images were captured by a CELENA S Digital Imaging System (20×). Scale bars = 100 μm. (**D**) Antimelanogenic effect of EGTA was evaluated by measuring extracellular (left) and intracellular (right) melanin contents. The results represent the average of data obtained from three independent experiments and are expressed as the mean ± standard error of the mean (SEM) (^###^ *p* < 0.001 and ^#^ *p* < 0.05 vs. untreated cells; *** *p* < 0.001, ** *p* < 0.01, and * *p* < 0.05 vs. α-MSH-treated cells). GABA, gamma-aminobutyric acid; α-MSH, α-melanocyte-stimulating hormone; cAMP, cyclic adenosine monophosphate (cAMP), CREB, cAMP response element-binding protein; EGTA, ethylene glycol-bis(2-aminoethylether)-*N,N,Nʹ,Nʹ*-tetraacetic acid.

As the interaction of α-MSH with MC1R initiated the melanogenic signaling pathway through the cAMP–CREB–*MITF* axis [3], cAMP levels were measured in B16F10 melanoma cells. As shown in Figure 4A, quantification of cAMP revealed that α-MSH significantly increased cAMP levels from 13.1 ± 0.5 to 155.3 ± 2.8 pg/mL. However, GABA inhibited α-MSH-induced cAMP levels, determined as 125.3 ± 2.8 pg/mL, 80.8 ± 2.9 pg/mL, and 44.2 ± 3.7 pg/mL at 5, 10, and 20 mM, respectively. We then evaluated the inhibitory effect of GABA on α-MSH-induced CREB phosphorylation. Under α-MSH stimulation, GABA effectively inhibited CREB phosphorylation in a concentration-dependent manner (Figure 4B). Previous studies have also revealed that CREB phosphorylation results in Ca^2+^ release from intracellular stores, stimulating cAMP production [5,22]. In the present study, staining of cytosolic Ca^2+^ using a Ca^2+^-sensitive probe, Fluo-4 AM, revealed that α-MSH-induced intracellular Ca^2+^ levels were markedly inhibited by GABA treatment when compared with the levels observed in the presence of ethylene glycol-bis(2-aminoethylether)-*N,N,Nʹ,Nʹ*-tetraacetic acid (EGTA), a well-known Ca^2+^-chelating agent (Figure 3C). Next, to evaluate the significance of increased Ca^2+^ levels in α-MSH-induced melanogenesis, the melanin content was measured after EGTA pretreatment. We observed that EGTA significantly inhibited α-MSH-induced extracellular (from 324.2% ± 9.9% to 202.5% ± 9.1%; Figure 4D) and intracellular (from 207.7% ± 6.7% to 136.6 ± 8.3%; Figure 4F) melanin contents. These findings indicate that GABA inhibits α-MSH-induced melanogenesis by inhibiting the cAMP–CREB axis and Ca^2+^ signaling pathways.

### 2.5. GABA_A_ Receptor Is Responsible for the Antimelanogenic Properties of GABA

**Figure 5:**
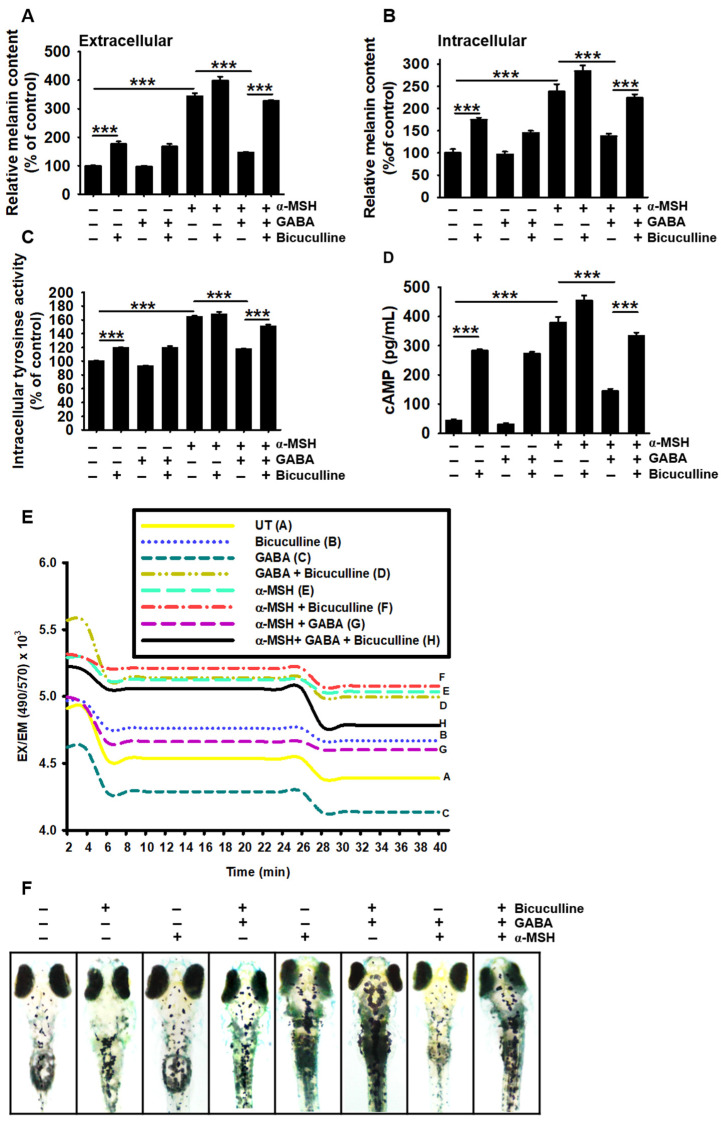
Bicuculline reverses the antimelanogenic properties of GABA in B16F10 melanoma cells and zebrafish larvae. B16F10 melanoma cells (5 × 10^4^ cells/mL) were pretreated with 10 µM (+)-bicuculline for 2 h prior to treatment with 20 mM GABA and 500 ng/mL α-MSH. (**A**) Extracellular and (**B**) intracellular melanin contents were measured. (**C**) Intracellular *tyrosinase* activity was measured 72 h after treatment with GABA and α-MSH, and (**D**) cAMP level was determined at 15 min. (**E**) The cells were stained with 1 μM Fluo-4 AM for 10 min after treatment with GABA and/or α-MSH for 1 h, and fluorescence intensity was measured using a luminometer. (**F**) Three days post-fertilized zebrafish larvae were pretreated with 10 µM bicuculline for 2 h before treatment with 50 mM GABA and 1 µg/mL α-MSH for an additional 72 h. Images were captured using a stereomicroscope (4×). The results represent the average of data obtained from three independent experiments and are expressed as the mean ± standard error of the mean (SEM) (*** *p* < 0.001). GABA, gamma-aminobutyric acid; α-MSH, α-melanocyte-stimulating hormone; cAMP, cyclic adenosine monophosphate.

The Cancer Genome Atlas (TCGA) revealed the presence of the GABA_A_ and GABA_B_ receptors in B16F10 melanoma cells [23,24]. Accordingly, we investigated the possible involvement of GABA receptors in GABA-mediated antimelanogenic properties. Treatment with a GABA_A_ receptor antagonist, bicuculline, significantly increased extracellular and intracellular melanin contents in B16F10 melanoma cells to 174.4% ± 5.1% and 176.5% ± 14.7%, respectively, when compared with those in the untreated cells (Figure 5A,B). In addition, bicuculline reversed GABA-induced antimelanogenic effects by increasing extracellular (from 146.2% ± 2.2% to 327.5% ± 3.2%) and intracellular (from 137.5% ± 5.8% to 223.9% ± 7.9%) melanin contents. Bicuculline also increased intracellular *tyrosinase* activity to 120.5% ± 0.9% (Figure 5C); under α-MSH stimulation (168.4% ± 4.5%), the GABA-mediated reduction in *tyrosinase* activity was reversed (from 110.7% ± 1.3% to 150.5% ± 2.9%) with treatment of bicuculline. Furthermore, bicuculline significantly upregulated cAMP levels from 42.6 ± 5.6 to 282.6 ± 5.6 pg/mL in B16F10 melanoma cells and potently reversed the GABA-induced decrease in cAMP levels (from 144.74 ± 7.3 to 333.64 ± 11.47 pg/mL) in α-MSH-treated B16F10 cells (Figure 5D). Moreover, GABA induced lower intracellular Ca^2+^ levels than those in untreated B16F10 melanoma cells; however, bicuculline markedly increased Ca^2+^ levels (Figure 5E). In α-MSH-stimulated B16F10 melanoma cells, the GABA-induced decrease in intracellular Ca^2+^ levels was markedly elevated following bicuculline treatment, suggesting that the GABA–GABA_A_ receptor axis downregulates intracellular Ca^2+^ levels. Bicuculline treatment also upregulated hyperpigmentation in α-MSH-stimulated zebrafish larvae, concomitant with the reversal of GABA-mediated antimelanogenic properties (Figure 5F); this indicates that GABA inhibits α-MSH-induced melanogenesis by downregulating the GABA_A_ receptor-mediated reduction in intracellular cAMP and Ca^2+^ levels.

### 2.6. GABA_B_ Receptor Regulates GABA-Mediated Antimelanogenic Properties

**Figure 6:**
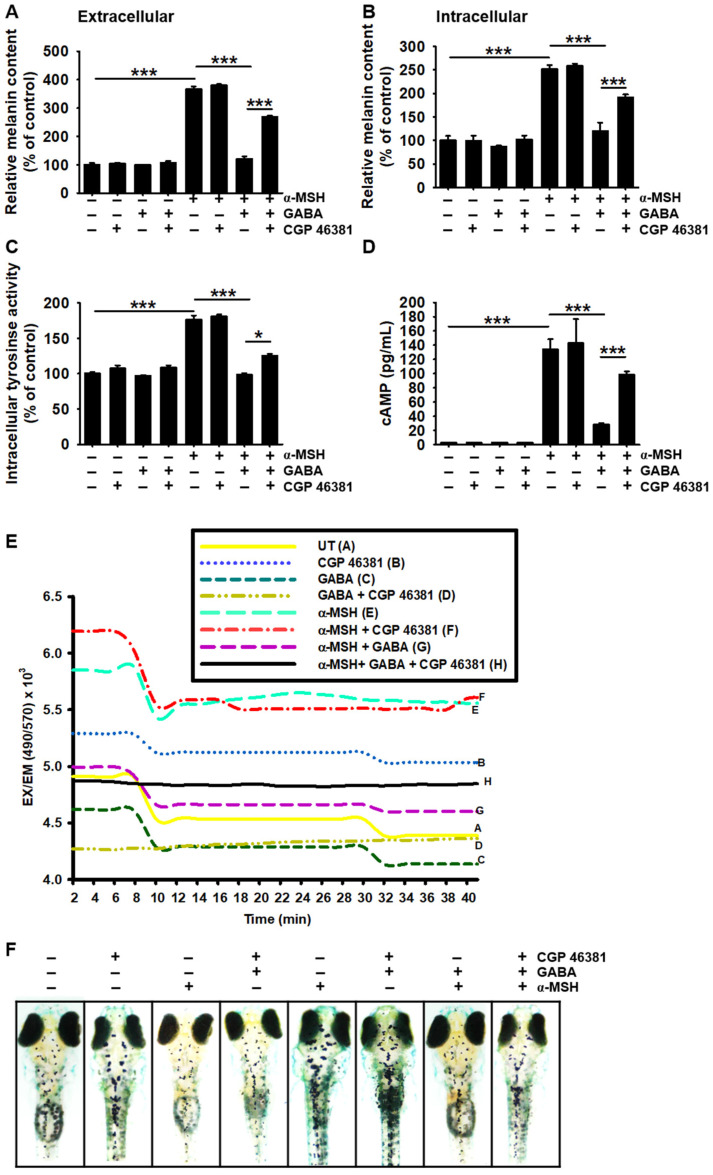
CGP 48381 reverses GABA-induced antimelanogenic effects in B16F10 melanoma cells and zebrafish larvae. B16F10 melanoma cells (5 × 10^4^ cells/mL) were pretreated with 10 µM CGP 46381 for 2 h prior to treatment with 20 mM GABA and 500 ng/mL α-MSH. Extracellular (**A**) and intracellular (**B**) melanin contents were measured. (**C**) Intracellular *tyrosinase* activity was measured 72 h after treatment with GABA and α-MSH, and (**D**) cAMP level was determined at 15 min. (**E**) The cells were stained with 1 μM Fluo-4 AM for 10 min after treatment with GABA and/or α-MSH for 1 h, and fluorescence intensity was measured for 40 min using a luminometer. (**F**) Three days post-fertilized zebrafish larvae were pretreated with 10 µM CGP 46381 for 2 h before treatment with 50 mM GABA and/or 1 µg/mL α-MSH for an additional 72 h. Images were captured using a stereomicroscope (4×). The results represent the average of data obtained from three independent experiments and are expressed as the mean ± standard error of the mean (SEM) (*** *p* < 0.001 and * *p* < 0.01). GABA, gamma-aminobutyric acid; α-MSH, α-melanocyte-stimulating hormone; cAMP, cyclic adenosine monophosphate.

Treatment with a GABA_B_ receptor antagonist, CGP 46381, demonstrated inhibitory activity similar to bicuculline during melanogenesis in α-MSH-treated B16F10 melanoma cells. CGP 46381 significantly reversed the antimelanogenic effect of GABA in α-MSH-treated B16F10 melanoma cells (from 121.2% ± 8.4% to 268.8% ± 5.1% of extracellular (Figure 6A) and from 119.9% ± 18.2% to 191.8% ± 7.9% of intracellular melanin content (Figure 6B)). Nevertheless, extracellular and intracellular melanin contents were not upregulated in the presence of CGP 46381 itself when compared with those observed in untreated cells. Additionally, CGP 46381 did not increase intracellular *tyrosinase* activity (Figure 6C) and cAMP levels (Figure 6D); however, during α-MSH stimulation, CGP 46381 significantly reversed GABA-induced inhibition of intracellular *tyrosinase* activity and cAMP levels (from 98.3% ± 2.5% to 125.4% ± 2.5% and from 28.1 ± 3.4 to 98.3 ± 8.0 pg/mL, respectively). We then evaluated the effect of CGP 46381 on intracellular Ca^2+^ accumulation and observed that CGP 46381 itself increased intracellular Ca^2+^ levels and reversed GABA-mediated inhibition of Ca^2+^ levels in α-MSH-stimulated B16F10 cells (Figure 6E). Finally, zebrafish larvae were pretreated with CGP 46381 before exposure to GABA and/or α-MSH. We observed that CGP 46381 markedly reversed GABA-mediated antimelanogenic effects (Figure 6F). These results indicate that the GABA_B_ receptor regulates GABA-mediated antimelanogenic properties through Ca^2+^-dependent signaling pathways.

## 3. Discussion

GABA is a critical non-nutritive amino acid that functions as an inhibitory neurotransmitter in the central nervous system [8]. Recent clinical studies have revealed that dietary GABA supplementation is associated with health benefits, such as reduced heart rate variability and salivary chromogranin A [25], diminished physiological and physical stress induced by occupational fatigue [26], and alleviated mental task-induced stress [27]. However, whether GABA regulates melanogenesis has not been previously elucidated. In the present study, we determined that GABA negatively regulates α-MSH-induced melanin biosynthesis through the GABA_A_ and GABA_B_ receptors, mediated by subsequent downregulation of the cAMP–CREB–*MITF*–*tyrosinase* axis and intracellular Ca^2+^ levels.

Ca^2+^ is a fundamental secondary messenger that can dictate multiple cell signaling pathways through GPCRs such as MC1R [28]. The interaction between α-MSH and MC1R results in the release of ER luminal Ca^2+^ into the cytosol [29], which, in turn, enhances melanogenesis in melanocytes, concurrent with the AC–cAMP–CREB–*MITF*–*tyrosinase* axis, and promotes the release of melanosomes containing melanin into the extracellular space through melanocyte dendrites [30]; this induces melanosome phagocytosis by keratinocytes and melanin dispersion around the nucleus of keratinocytes [31]. Ca^2+^ also activates PKCβ-mediated phosphorylation and activation of *tyrosinase*, leading to melanogenesis [32,33]. Therefore, upregulation of intracellular Ca^2+^ stimulates melanogenesis in melanocytes and promotes melanosome transfer to keratinocytes, indicating that the Ca^2+^ signaling pathway plays an important role in modulating melanin production and dispersion. In the present study, we demonstrated that an increase in intracellular Ca^2+^ is pivotal for α-MSH-induced melanogenesis, and GABA inhibits α-MSH-induced melanogenesis by downregulating intracellular Ca^2+^ levels in B16F10 melanoma cells and zebrafish larvae. Additionally, Buffey et al. [34] suggested that Ca^2+^ is involved in distinct melanogenesis steps; Ca^2+^ induces an inhibitory effect on *tyrosinase* activity in unstimulated cells while activating *tyrosinase* activity in the presence of cAMP. Therefore, whether the GABA interaction with the GABA_A_ and GABA_B_ receptors regulates intracellular Ca^2+^ levels in melanogenesis needs to be determined.

Recent studies have shown that GABA_A_ receptor antagonists potently increase melanocyte production from melanocyte stem cells in zebrafish larvae, suggesting that the GABA_A_ receptor maintains melanocyte stem cell quiescence [35,36]. In addition, Allen et al. [36] reported that pharmacological activation of the GABA_A_ receptor inhibits melanocyte regeneration, which indicates that the GABA_A_ receptor signaling pathway induces antimelanogenesis by maintaining melanocyte stem cell quiescence. In the present study, bicuculline, a GABA_A_ receptor antagonist, significantly increased extracellular and intracellular melanin contents, along with a concomitant increase in *tyrosinase* activity and upregulation of intracellular Ca^2+^ levels. Additionally, GABA downregulated α-MSH-induced melanogenesis by decreasing intracellular Ca^2+^ in B16F10 melanoma cells and zebrafish larvae; however, in the presence of bicuculline, the antimelanogenic effect of GABA significantly decreased, accompanied by high intracellular Ca^2+^ levels. Therefore, our findings indicate that GABA activates the GABA_A_ receptor and subsequently inhibits α-MSH-induced melanogenesis by inhibiting intracellular Ca^2+^ levels. However, a previous study revealed that GABA transiently upregulated intracellular Ca^2+^ levels in mouse dorsal root ganglion neurons through high voltage-activated channels, and a GABA_A_ receptor antagonist, (+)-bicuculline, antagonized the GABA-induced increase in intracellular Ca^2+^ [37]. In contrast, Mestdagh and Wűlfert [38] determined that (+)-bicuculline increased Ca^2+^ transients in cerebellar granule cells through non-GABA_A_ receptor-associated mechanisms [38]. Furthermore, Heidelberger and Matthews [39] reported that GABA did not cause an overall suppression of Ca^2+^ currents in single synaptic terminals, and, in particular, picrotoxin, a GABA_A_ receptor antagonist, was found to induce sustained intracellular Ca^2+^ levels in the presence of GABA. Additionally, the GABA_B_ receptor is a GABAergic GPCR, whose activation is directly linked with cAMP and intracellular Ca^2+^ in B16F10 melanoma cells [24]. In the present study, we observed that GABA inhibited α-MSH-induced melanogenesis, with a concurrent decrease in intracellular Ca^2+^ levels, and the effects were diminished in the presence of the GABA_B_ receptor antagonist CGP 46381. In particular, CGP 46381 itself moderately increased intracellular Ca^2+^ levels when compared with the untreated cells and did not contribute to the α-MSH-induced Ca^2+^ increase. In contrast to our findings, CGP 46381 reportedly inhibited baclofen-induced intracellular Ca^2+^ signaling in non-neuronal cells [40]. Although we determined that GABA inhibited α-MSH-induced melanogenesis through the GABA_A_ and GABA_B_ receptors, it remains unclear whether, as it was previously shown, intracellular Ca^2+^ levels in the presence of GABA and GABA receptors can be attributed to differences between melanocytes and neuronal cells, or K^+^ and Cl^-^ concentrations. In addition, whether Ca^+^ is indispensable for GABA-mediated melanogenesis needs to be comprehensively investigated. Reportedly, the GABA_C_ receptor also increases intracellular Ca^2+^ levels in human retinal pigment epithelial cells [41] and promotes cAMP-dependent PKA activation in neuronal cells [42]. However, inhibition of the GABA_C_ receptor using TPMPA, a selective GABA_C_ receptor antagonist, had no effect on melanogenesis in the presence of α-MSH or GABA (data not shown), indicating that the GABA_C_ receptor is not involved in melanogenesis.

## 4. Materials and Methods

### 4.1. Reagents and Antibodies

GABA, phenylthiourea (PTU), α-MSH, 3-(4,5-Dimethylthiazol-2-yl)-2,5-diphenyl-tetrazolium bromide (MTT), and EGTA were purchased from Sigma-Aldrich (St. Louis, MO, USA). Dulbecco’s Modified Eagle Medium (DMEM), fetal bovine serum (FBS), and antibiotic mixture were purchased from WELGENE (Gyeongsan, Gyeongsangbuk-do, Republic of Korea). Bicuculline and CGP 46381 were purchased from Tocris Bioscience (Bristol, United Kingdom). Antibodies against CREB-1 (sc-377154, 43 kDa), phospho (p)-CREB (sc-81486, 43 kDa), *MITF* (sc-71588, 60 kDa), *tyrosinase* (sc-20035, 84 kDa), β-actin (sc-69879, 43 kDa), and peroxidase-labeled anti-mouse immunoglobulins (sc-516102) were purchased from Santa Cruz Biotechnology (Santa Cruz, CA, USA). Peroxidase-labeled anti-rabbit immunoglobulins were obtained from KOMA Biotechnology (Seoul, Republic of Korea). Fluo-4 AM was obtained from Thermo Fisher Scientific (Waltham, MA, USA). All other chemicals were purchased from Sigma-Aldrich.

### 4.2. Cell Culture and Viability

B16F10 melanoma cells were obtained from the American Type Culture Collection (Manassas, VA, USA). The cells were cultured in DMEM supplemented with 10% heat-inactivated FBS and antibiotic mixture at 37°C in a 5% CO_2_ humidified incubator. To measure cell viability, B16F10 melanoma cells were seeded at a density of 5 × 10^4^ cells/mL in 96-well plates and then treated with GABA (0–100 mM) for 96 h. The cells were treated with MTT (0.5 mg/mL) solution for 1 h at 37°C, and formazan was dissolved with dimethyl sulfoxide (DMSO). Absorbance was measured at 540 nm using a microplate spectrophotometer (BioTek Instruments Inc., Winooski, VT, USA). In a parallel experiment, the images of the cell were captured using phase-contrast microscopy (MACROTECH, Goyang, Gyeonggido, Republic of Korea).

### 4.3. Flow Cytometry Analysis

B16F10 melanoma cells (5 × 10^4^ cells/mL) were cultured in 6-well plates and treated with the indicated concentrations of GABA (0–100 mM) for 96 h. The cells were washed with ice-cold phosphate-buffered saline (PBS) and incubated for 5 min using a Muse Cell Count and Viability Kit (Luminex, Austin, TX, USA). The population of viable (%) and dead cells (%) was analyzed by a Muse Cell Analyzer (Luminex).

### 4.4. Measurement of Extracellular and Intracellular Melanin Content

B16F10 melanoma cells (5 × 10^4^ cells/mL) were cultured in 6-well plates and treated with the indicated concentrations of GABA (0–20 mM) in the presence or absence of 500 ng/mL α-MSH for 96 h. In a parallel experiment, bicuculline or CGP 46381 was pretreated for 2 h and subsequently treated with GABA and/or α-MSH. Cell-free culture media were used for the quantification of extracellular melanin content. The cell pellets were washed with ice-cold PBS and dissolved with 1 M NaOH containing 10% DMSO at 90°C for 60 min. Extracellular and intracellular melanin contents were measured at 405 nm using a microplate spectrophotometer (BioTek Instruments Inc.).

### 4.5. cAMP Enzyme-Linked Immunosorbent Assay (ELISA)

B16F10 melanoma cells (5 × 10^4^ cells/mL) were cultured in serum-free DMEM media for 24 h and pretreated with the indicated concentrations of GABA (0–20 mM) in the presence or absence of 500 ng/mL α-MSH for 15 min [43]. In a parallel experiment, bicuculline or CGP 46381 was pretreated 2 h before treatment with GABA and/or α-MSH. The total amount of proteins in the cell lysate was quantified using Bio-Rad Protein Assay Reagents (Bio-Rad, Hercules, CA, USA), and a colorimetric ELISA kit (Cell Biolabs Inc., San Diego, CA, USA) was performed to quantify the intracellular cAMP levels. Finally, the absorbance was measured at 450 nm, and the amount of cAMP was calculated based on the cAMP standard curve.

### 4.6. Reverse Transcription-Polymerase Chain Reaction (RT-PCR)

B16F10 melanoma cells (5 × 10^4^ cells/mL) were treated with the indicated concentrations of GABA (0–20 mM) in the presence or absence of 500 ng/mL α-MSH for 48 h. Total RNA was extracted using an easy-BLUE RNA Extraction Kit (iNtRON Biotechnology, Sungnam, Korea), and cDNA was synthesized using MMLV reverse transcriptase (Bioneer, Daejeon, Korea). *MITF*, *tyrosinase*, and glyceraldehyde 3-phosphate dehydrogenase (*GAPDH*) expression was evaluated by amplifying the cDNA using EzWay PCR Ready Mix (KOMA Biotechnology) with specific primers (Table 1) [44].

### 4.7. Western Blotting

B16F10 melanoma cells (5 × 10^4^ cells/mL) were treated with the indicated concentrations of GABA (0–20 mM) in the presence or absence of 500 ng/mL α-MSH for 72 h. Total protein was extracted using a PRO-PREP Protein Extraction Solution Kit (iNtRON Biotechnology), and total proteins were quantified using a Bio-Rad Protein Assay Kit (Bio-Rad). Western blotting was performed to detect the expression of *MITF*, *tyrosinase*, and pCREB according to our previous study [44]. β-Actin was used as an internal control.

### 4.8. Measurement of Intracellular Tyrosinase Activity

Intracellular *tyrosinase* activity was measured using a previously described method [45]. Briefly, B16F10 melanoma cells (5 × 10^4^ cells/mL) were treated with 20 mM GABA in the presence or absence of 500 ng/mL α-MSH for 72 h. In a parallel experiment, bicuculline or CGP 46381 was pretreated for 2 h in the presence or absence of GABA and/or α-MSH. Then, the cells were lysed with PBS containing 1% Triton X-100 by freezing at −20 °C for 2 h and disrupted by thawing at room temperature. Total protein was quantified with Bio-Rad Protein Assay Reagents (Bio-Rad), and an equal amount of protein was mixed with 90 μL of 5 mM L-dihydroxyphenylalanine (DOPA) at 37 °C for 30 min and the absorbance at 405 nm was measured.

### 4.9. Staining of Intracellular Tyrosinase

B16F10 melanoma cells (5 × 10^4^ cells/mL) were cultured in 3% gelatin-coated cover slips and treated with 20 mM GABA in the presence or absence of 500 ng/mL α-MSH for 72 h. The cells were fixed with 4% paraformaldehyde for 30 min and incubated with 0.1% L-DOPA in 1× PBS and 20 μM copper sulfate at 37 °C for 2 h. The images were captured using phase-contrast microscopy (MACROTECH).

### 4.10. Kinetics Scan of Intracellular Ca^2+^ Changes

Intracellular Ca^2+^ changes were measured according to a previously described method with a slight modification [22]. Briefly, B16F10 murine melanoma cells (5 × 10^4^ cells/mL) were grown in a 12-well plate and pretreated with bicuculline or CGP 46381 for 2 h prior to treatment with 20 mM GABA and/or 500 ng/mL α-MSH. Then, the cells were trypsinized and probed with a Ca^2+^-sensitive probe, Fluo-4 AM (1 μM) in calcium buffer (135 mM NaCl, 4.5 mM KCl, 1.5 mM CaCl_2_, 0.5 mM MgCl_2_, 10 mM HEPES, and 5.6 mM glucose, pH 7.4), for 10 min. Kinetics scans were performed for 40 min to assess the time course of Ca^2+^ fluorescence changes at EX/EM 490/570 using a GloMax 96 Microplate Luminometer (Promega, Madison, WI, USA).

### 4.11. Imaging for Intracellular Ca^2+^

B16F10 melanoma cells were cultured in 8-well chamber slides and treated with 20 mM GABA or 2 mM EGTA in the presence and absence of 500 ng/mL α-MSH for 1 h. Then, the cells were washed with PBS and probed with 1 μM Fluo-4 AM in calcium buffer for 10 min. The images of the cells were captured using a CELENA S Digital Imaging System (Logos Biosystems, Anyang, Gyeonggi-do, Korea).

### 4.12. Evaluation of Antimelanogenic Properties of GABA in Zebrafish Larvae

The zebrafish was raised and handled according to standard guidelines of the Animal Care and Use Committee of Jeju National University (Jeju Special Self-Governing Province, Korea; approval No.: 2021-0008). All methods were carried out in accordance with the approved guidelines [46]. Eggs were collected after the natural spawning of an inbred AB strain of zebrafish and raised in E3 embryo media containing 2 mg/L methylene blue. Zebrafish embryos after 2 days post-fertilization (2 dpf) were treated with 200 nM PTU for 24 h and replaced the media containing the indicated concentrations of GABA (0–50 mM) and/or 1 μg/mL α-MSH for 3 days (6 dpf). In a parallel experiment, bicuculline and CGP 46381 were treated for 2 h prior to treatment with GABA and/or α-MSH. The images of the larvae were taken at 6 dpf using stereomicroscopy (Olympus, Tokyo, Japan). Heart rates were manually counted for 2 min and expressed as beats/min to evaluate the cardiotoxicity.

### 4.13. Statistical Analysis

All the Western blots and RT-PCR bands were quantified by ImageJ 1.50i (National Institute of Health, Manassas, VA, USA, www.imagej.net, accessed on 15 June 2021) and then statistically analyzed by Sigma plot 12.0 (Systat Software, San Jose, CA, USA, www.systatsoftware.com, accessed on 15 June 2021). All data represented the mean of at least three independent experiments. Significant differences were determined using a Student *t*-test and an unpaired one-way analysis of variance (ANOVA) test with Bonferroni correction. Statistical significance was set at *** and ^###^ *p* < 0.001, ** and ^##^ *p* < 0.01, and * and ^#^ *p* < 0.05.

## 5. Conclusions

In this study, we determined that, under α-MSH stimulation, the GABA_A_ and GABA_B_ receptors are associated with the antimelanogenic properties of GABA, accompanied by the inhibition of the canonical melanogenic signaling pathway and intracellular Ca^2+^ levels. Our findings may contribute to the potential use of GABA for the development of antimelanogenic agents. Nevertheless, whether the Ca^2+^ signaling pathway is strongly associated with the antimelanogenic properties of GABA should be evaluated, in detail, by targeting the GABA_A_ and GABA_B_ receptors.

## Figures and Tables

**Table 1:** Primers and PCR conditions.

Gene *	Primer Sequence (5′-3′)	Size	T_m_	Cycle No.
*MITF*	F: 5′-CCCGTCTCTGGAAACTTGATCG-3′	767 bp	60 °C	27
	R: 5′-CTGTACTCTGAGCAGCAGGTC-3′			
*Tyrosinase*	F: 5′-GTCGTCACCCTGAAAATCCTAACT-3′	110 bp	60 °C	27
	R: 5′-CATCGCATAAAACCTGATGGC-3′			
*GAPDH*	F: 5′-ACCACAGTCCATGCCATCAC-3′	480 bp	60 °C	25
	R: 5′-CACCACCCTGTTGCTGTAGC-3′			

F, forward primer; R, reverse primer; bp; base pair; T_m_, melting temperature. * *MITF*, microphthalmia-associated transcription factor; *GAPDH*, glyceraldehyde 3-phosphate dehydrogenase.

## Data Availability

All data are contained within the article.

## References

[1] Brenner M., Hearing V.J. (2008). The protective role of melanin against UV damage in human skin. Photochem. Photobiol..

[2] D’Agostino G., Diano S. (2010). Alpha-melanocyte stimulating hormone: Production and degradation. J. Mol. Med..

[3] García-Borrón J.C., Abdel-Malek Z., Jiménez-Cervantes C. (2014). MC1R, the cAMP pathway, and the response to solar UV: Extending the horizon beyond pigmentation. Pigment Cell Melanoma Res..

[4] Motiani R.K., Tanwar J., Raja D.A., Vashisht A., Khanna S., Sharma S., Srivastava S., Sivasubbu S., Natarajan V.T., Gokhale R.S. (2018). STIM1 activation of adenylyl cyclase 6 connects Ca^2+^ and cAMP signaling during melanogenesis. EMBO J..

[5] Cooper D.M. (2015). Store-operated Ca²⁺-entry and adenylyl cyclase. Cell Calcium.

[6] D’Mello S.A.N., Finlay G.J., Baguley B.C., Askarian-Amiri M.E. (2016). Signaling Pathways in Melanogenesis. Int. J. Mol. Sci..

[7] Cestari T.F., Dantas L.P., Boza J.C. (2014). Acquired hyperpigmentations. An. Bras. Dermatol..

[8] Nuss P. (2015). Anxiety disorders and GABA neurotransmission: A disturbance of modulation. Neuropsychiatr. Dis. Treat..

[9] Diana M., Quílez J., Rafecas M. (2014). Gamma-aminobutyric acid as a bioactive compound in foods: A review. J. Funct. Foods.

[10] Lin S.-D., Mau J.-L., Hsu C.-A. (2012). Bioactive components and antioxidant properties of γ-aminobutyric acid (GABA) tea leaves. Food Sci. Technol..

[11] Roohinejad S., Omidizadeh A., Mirhosseini H., Saari N., Mustafa S., Mohd Yusof R., Meor Hussin A.S., Hamid A., Abd Manap M.Y. (2010). Effect of pre-germination time of brown rice on serum cholesterol levels of hypercholesterolaemic rats. J. Sci. Food Agric..

[12] Tian J., Lu Y., Zhang H., Chau C.H., Dang H.N., Kaufman D.L. (2004). γ-Aminobutyric acid inhibits T cell autoimmunity and the development of inflammatory responses in a mouse type 1 diabetes model. J. Immunol..

[13] Oh S.-H., Soh J.-R., Cha Y.-S. (2003). Germinated brown rice extract shows a nutraceutical effect in the recovery of chronic alcohol-related symptoms. J. Med. Food.

[14] Smith T.A. (2001). Type A gamma-aminobutyric acid (GABAA) receptor subunits and benzodiazepine binding: Significance to clinical syndromes and their treatment. Br. J. Biomed. Sci..

[15] Chebib M., Johnston G.A. (1999). The ’ABC’ of GABA receptors: A brief review. Clin. Exp. Pharmacol. Physiol..

[16] Sigel E., Steinmann M.E. (2012). Structure, function, and modulation of GABA_A_ receptors. J. Biol. Chem..

[17] Cheng T., Wallace D.M., Ponteri B., Tuli M. (2018). Valium without dependence? Individual GABA_A_ receptor subtype contribution toward benzodiazepine addiction, tolerance, and therapeutic effects. Neuropsychiatr. Dis. Treat..

[18] Olsen R.W., Sieghart W. (2009). GABA A receptors: Subtypes provide diversity of function and pharmacology. Neuropharmacology.

[19] Sieghart W., Enna S.J., Möhler H. (2007). Subunit composition and structure of GABA_A_-receptor subtypes. The GABA Receptors.

[20] Olsen R.W., Gordey M., Endo M., Kurachi Y., Mishina M. (2000). GABAA Receptor Chloride Ion Channels. Pharmacology of Ionic Channel Function: Activators and Inhibitors.

[21] Terunuma M. (2018). Diversity of structure and function of GABA_B_ receptors: A complexity of GABA(B)-mediated signaling. Proc. Jpn. Acad. Ser. B Phys. Biol. Sci..

[22] Wood J.M., Hoogduijn M.J., Smit N.P., Thody A.J., Van Der Laarse A., Van Nieuwpoort A.F. (2003). Melanin has a role in Ca^2+^ homeostasis in human melanocytes. Pigment Cell Res..

[23] Pomeranz Krummel D.A., Nasti T.H., Kaluzova M., Kallay L., Bhattacharya D., Melms J.C., Izar B., Xu M., Burnham A., Ahmed T. (2020). Melanoma cell intrinsic GABA_A_ receptor enhancement potentiates radiation and immune checkpoint inhibitor response by promoting direct and T cell-mediated antitumor activity. Int. J. Radiat. Oncol. Biol. Phys..

[24] Shen W., Li Y., Li B., Zheng L., Xie X., Le J., Lu Y., Li T., Chen F., Jia L. (2019). Downregulation of KCTD12 contributes to melanoma stemness by modulating CD271. Cancer Biol. Med..

[25] Nakamura H., Takishima T., Kometani T., Yokogoshi H. (2009). Psychological stress-reducing effect of chocolate enriched with γ-aminobutyric acid (GABA) in humans: Assessment of stress using heart rate variability and salivary chromogranin A. Int. J. Food Sci. Nutr..

[26] Kanehira T., Nakamura Y., Nakamura K., Horie K., Horie N., Furugori K., Sauchi Y., Yokogoshi H. (2011). Relieving occupational fatigue by consumption of a beverage containing γ-amino butyric acid. J. Nutr. Sci. Vitaminol..

[27] Arredouani A., Yu F., Sun L., Machaca K. (2010). Regulation of store-operated Ca^2+^ entry during the cell cycle. J. Cell Sci..

[28] Gudermann T., Bader M. (2015). Receptors, G proteins, and integration of calcium signalling. J. Mol. Med..

[29] Rodrigues A.R., Almeida H., Gouveia A.M. (2015). Intracellular signaling mechanisms of the melanocortin receptors: Current state of the art. Cell. Mol. Life Sci..

[30] Carsberg C.J., Jones K.T., Sharpe G.R., Friedmann P.S. (1995). Intracellular calcium modulates the responses of human melanocytes to melanogenic stimuli. J. Dermatol. Sci..

[31] Ando H., Niki Y., Ito M., Akiyama K., Matsui M.S., Yarosh D.B., Ichihashi M. (2012). Melanosomes are transferred from melanocytes to keratinocytes through the processes of packaging, release, uptake, and dispersion. J. Investig. Dermatol..

[32] Park H.Y., Perez J.M., Laursen R., Hara M., Gilchrest B.A. (1999). Protein kinase C-beta activates *tyrosinase* by phosphorylating serine residues in its cytoplasmic domain. J. Biol. Chem..

[33] Park H.Y., Wu H., Killoran C.E., Gilchrest B.A. (2004). The receptor for activated C-kinase-I (RACK-I) anchors activated PKC-beta on melanosomes. J. Cell Sci..

[34] Buffey J.A., Edgecombe M., Mac Neil S. (1993). Calcium plays a complex role in the regulation of melanogenesis in murine B16 melanoma cells. Pigment Cell Res..

[35] Allen J.R., Skeath J.B., Johnson S.L. (2020). GABA-A receptor and mitochondrial TSPO signaling act in parallel to regulate melanocyte stem cell quiescence in larval zebrafish. Pigment Cell Melanoma Res..

[36] Allen J.R., Skeath J.B., Johnson S.L. (2019). Maintenance of melanocyte stem cell quiescence by GABA-A signaling in larval zebrafish. Genetics.

[37] Yokogawa T., Kim S.U., Krieger C., Puil E. (2001). Analysis of GABA(A)- and GABA(B)-receptor mediated effects on intracellular Ca^2+^ in DRG hybrid neurones. Br. J. Pharmacol..

[38] Mestdagh N., Wulfert E. (1999). Bicuculline increases Ca^2+^ transients in rat cerebellar granule cells through non-GABA_A_ receptor associated mechanisms. Neurosci. Lett..

[39] Heidelberger R., Matthews G. (1991). Inhibition of calcium influx and calcium current by gamma-aminobutyric acid in single synaptic terminals. Proc. Natl. Acad. Sci. USA.

[40] Cheng Z.Y., Wang X.P., Schmid K.L., Han X.G. (2014). GABAB1 and GABAB2 receptor subunits co-expressed in cultured human RPE cells regulate intracellular Ca^2+^ via G_i/o_-protein and phospholipase C pathways. Neuroscience.

[41] Strøbaek D., Jørgensen T.D., Christophersen P., Ahring P.K., Olesen S.P. (2000). Pharmacological characterization of small-conductance Ca^2+^-activated K^+^ channels stably expressed in HEK 293 cells. Br. J. Pharmacol..

[42] Yang L., Nakayama Y., Hattori N., Liu B., Inagaki C. (2008). GABA_C_-receptor stimulation activates cAMP-dependent protein kinase via A-kinase anchoring protein 220. J. Pharmacol. Sci..

[43] Isoldi M.C., Pereira E.A., Visconti M.A., Castrucci A.M. (2004). The role of calcium, calcium-activated K^+^ channels, and tyrosine/kinase in psoralen-evoked responses in human melanoma cells. Braz. J. Med. Biol. Res..

[44] Molagoda I.M.N., Karunarathne W., Park S.R., Choi Y.H., Park E.K., Jin C.Y., Yu H., Jo W.S., Lee K.T., Kim G.Y. (2020). GSK-3β-targeting fisetin promotes melanogenesis in B16F10 melanoma cells and zebrafish larvae through β-catenin activation. Int. J. Mol. Sci..

[45] Molagoda I.M.N., Choi Y.H., Lee S., Sung J., Lee C.R., Lee H.G., Lim J., Lee K.-J., Jeon Y.-J., Ma J. (2020). Ethanolic extract of *Hippocampus abdominalis* exerts anti-melanogenic effects in B16F10 melanoma cells and zebrafish larvae by activating the ERK signaling pathway. Cosmetics.

[46] Percie du Sert N., Ahluwalia A., Alam S., Avey M.T., Baker M., Browne W.J., Clark A., Cuthill I.C., Dirnagl U., Emerson M. (2020). Reporting animal research: Explanation and elaboration for the ARRIVE guidelines 2.0. PLoS Biol..

